# Cytotaxonomy of the subgenus Artibeus (Phyllostomidae, Chiroptera) by characterization of species-specific markers

**DOI:** 10.3897/CompCytogen.v6i1.1510

**Published:** 2012-01-24

**Authors:** Marcela Maria Pereira de Lemos Pinto, Merilane da Silva Calixto, Maria José de Souza, Ana Paloma Tavares de Araújo, Alfredo Langguth, Neide Santos

**Affiliations:** 1 Departamento de Genética, Laboratório de Genética e Citogenética Animal, Universidade Federal de Pernambuco, Recife, PE, Brasil; 2 Departamento de Sistemática e Ecologia, Universidade Federal da Paraíba, João Pessoa, PB, Brasil

**Keywords:** C-banding, Ag-NOR, CMA_3_/DA/DAPI, Cytotaxonomy

## Abstract

The genus *Artibeus* represents a highly diverse group of bats from the Neotropical region, with four large species occurring in Brazil. In this paper, a comparative cytogenetic study was carried out on the species *Artibeus obscurus* Schinz, 1821, *Artibeus fimbriatus* Gray, 1838, *Artibeus lituratus* Olfers, 1818 and *Artibeus planirostris* Spix, 1823 that live sympatrically in the northeast of Brazil, through C-banding, silver staining and DNA-specific fluorochromes (CMA_3_ and DAPI). All the species had karyotypes with 2n=30,XX and 2n=31,XY_1_Y_2_, and FN=56. C-banding showed constitutive heterochromatin (CH) blocks in the pericentromeric regions of all the chromosomes and small CH blocks at the terminal region of pairs 5, 6, and 7 for all species. Notably, our C-banding data revealed species-specific autosomic CH blocks for each taxon, as well as different heterochromatic constitution of Y_2 _chromosomes of *Artibeus planirostris*. Ag-NORs were observed in the short arms of chromosomes 5, 6 and 7 in all species. The sequential staining AgNO_3_/CMA_3_/DA/DAPI indicated a positive association of CH with Ag-NORs and positive CMA_3 _signals, thus reflecting GC-richness in these regions in *Artibeus obscurus* and *Artibeus fimbriatus*. In this work it was possible to identify interespecific divergences in the Brazilian large *Artibeus* species using C-banding it was possible provided a suitable tool in the cytotaxonomic differentiation of this genus.

## Introduction

The genus *Artibeus* Leach, 1821 has been divided into two main groups based on body size. The species with larger body size have been classified as subgenus *Artibeus* and the species with smaller body size as the subgenus *Dermanura* Gervais, 1856. In addition,a new subgenus *Koopmania* Owen, 1991 was proposed by Owen (1987, 1991) to set apart one of the species, *Artibeus concolor* Peters, 1865.However, this subgenus was later disregarded by Van De Bussche et al. (1998) based on morphological, enzymatic, molecular and karyotypic analysis. The taxonomic classification proposed by Hoofer et al. (2008) recognized *Artibeus* as a distinct subgenus from *Dermanura*. Recently, Marchán-Rivadeneira et al. (2010) described the genus *Artibeus* as constituted by 11 large-body size species including *Artibeus concolor* as the basal taxon. Its distributional range is restricted to the Neotropical region. The genus is widely distributed from Mexico to northern Argentina, including the Antillean islands in the Caribbean (Simmons 2005).

The extensive similarity of morphometric characters, high degree of shape diversity and overlapping of natural habitats have hindered accurate identification of the large *Artibeus* along their distribution, particularly in the Neotropical region (Haynes and Lee 2004, Hollis 2005). A typical example is the northeastern region of Brazil where four species of the large *Artibeus* (*Artibeus obscurus* Schinz, 1821, *Artibeus fimbriatus* Gray, 1838, *Artibeus lituratus* Olfers, 1818 and *Artibeus planirostris* Spix, 1823) were formally recorded living in sympatry (Taddei et al. 1998, Araújo and Langguth 2010). In this region, similarity in morphometric measurements (e.g. cranial distances and external dimensions) and geographical variation of *Artibeus planirostris* are main reasons for confusing taxonomy (Guerrero et al. 2003, Simmons 2005).

Since the systematic classification of subgenus *Artibeus* remains subject of several discussions concerning phylogenetic relationships and actual taxonomic status of species, the use of complementary information may help to define species more precisely (Larsen et al. 2010). For other Mammalian groups, such as primates, felines and rodents, classical and molecular cytogenetic analysis have been successfully allied to taxonomic studies to identify species since chromosomes are not affected by adaptation process to different feeding niches as cranial and general gross anatomies (Granjon and Dobigny 2003, Garcia and Pessoa 2010).

In this work a karyotypic characterization of *Artibeus obscurus* Schinz, 1821, *Artibeus fimbriatus* Gray, 1838, *Artibeus lituratus* Olfers, 1818 and *Artibeus planirostris* Spix, 1823 from northeastern of Brazil was performed by the cytogenetic techniques – conventional analysis, C-banding, Ag-NOR and triple staining CMA_3_/DA/DAPI. The data were helpful to carry a comparative analysis of those species, in terms of interspecific differences, and also to provide a better identification of them.

## Material and methods

Based on literature (Handley 1989, Taddei et al. 1998), the following characters were used to diagnose the species: presence of fur on the forearms, structure of legs and interfemoral membrane; color of body, dorsal and ventral fur; facial stripes; form of nose leaf and its relationship with the upper lip; shape of the pre- and postorbital process and postorbital constriction and the presence or absence of the 3rd molar. The identification process also included the following 10 measurements: length of forearm, condylobasal length; length of maxillary tooth-row; length of lower tooth-row; length of mandible; breadth across upper canines; mastoidal breadth; zygomatic breadth; postorbital constriction; breadth across upper molars.

After identification, cytogenetic studies were carried out on 53 *Artibeus* specimens from the state of Pernambuco, northeastern Brazil. Voucher specimens are deposited in the Mammalian collection at the Department of Systematic and Ecology, Federal University of Paraíba, João Pessoa, Paraiba, Brazil. The specimens studied were six males and eight females of *Artibeus obscurus*; two males and four females of *Artibeus fimbriatus*; eight males and five females of *Artibeus planirostris*; ten males and ten females of *Artibeus lituratus* captured at different sites across the Pernambuco State: Igarassu (07°50'02"S, 34°54'21"W), Água Preta (08°42'27"S, 35°31'50"W), Rio Formoso (08°39'50"S, 35°09'32"W), Ipojuca (08°24'00"S, 35°03'45"W) and Recife (08°03'14"S, 34°52'51"W) (see also Appendix).

Metaphase spreads were obtained from bone marrow cells according to conventional procedures and staining with Giemsa. C-banding and silver staining were performed according to Sumner (1972) and Howell and Black (1980), respectively. Triple staining CMA_3_/DA/DAPI was carried out according to Santos and Souza (1998a).

For sequential staining (AgNO_3_/CMA_3_/DA/DAPI), the slides stained by silver nitrate were distained after photographing (Dos Santos Guerra 1991) and re-stained by CMA_3_/DA/DAPI. Photomicrographs were taken using Leica DMLB photomicroscope for C-banding and silver staining. Sequential staining images were captured by IM50 capture system.

## Results

All four species shared the same diploid number (2n=30, gap XX; 2n=31, gap XY_1_Y_2_) and fundamental number FN=56. Chromosomes were meta-submetacentric (1-4, 8-14), subtelocentric (5, 6, 7 and X) and two small acrocentric (Y_1_ and Y_2_). Except for the size of Y_1_ and Y_2_ chromosomes, it was not found any intraspecific variation between species analyzed with conventional staining.

**Figure 1.**
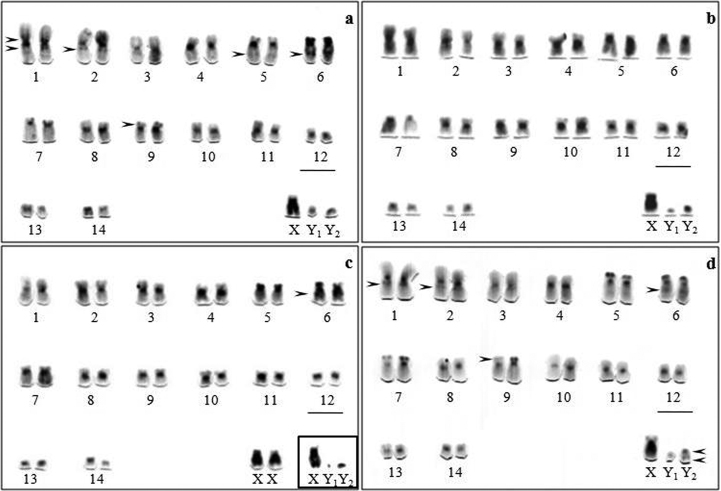
C-banding of *Artibeus obscurus* **(a)** *Artibeus fimbriatus* **(b)** *Artibeus lituratus* **(c)** and *Artibeus planirostris* **(d)** karyotypes. The arrowheads indicate a particular set of CH blocks in each species. Bar = 5 µm.

C-banding revealed constitutive heterochromatin (CH) in the pericentromeric region of all the autosomes and small heterochromatic blocks were observed in the terminal region of chromosome pairs 5, 6 and 7 (Fig. 1a–d). The karyotype of *Artibeus obscurus* (Fig. 1a) exhibited interstitial blocks in the short and long arms of pair 1, as well as in the long arms of pairs 2, 5, 6 and in the terminal region of the short arm of pair 9. The *Artibeus planirostris* karyotype (Fig. 1d) has the same CH pattern but lacks interstitial blocks in the short arm of chromosome 1. Absence of an interstitial block on the chromosome 6 distinguished karyotype of *Artibeus fimbriatus* from the other investigated karyotypes (Fig. 1). In all the material examined the long arms of the X chromosomes were more darkly stained when compared with the euchromatin of the autosomes. The Y_2_ appeared almost entirely heterochromatic in all species, except for *Artibeus planirostris* which showed pericentromeric and distal blocks (Fig. 1d). The pattern of the Y_1_ could not be determined with precision due to its punctiform size.

Table 1 shows exhibits the C-banding pattern in chromosomal complement in all species analyzed.

**Table 1.** Heterochromatin pattern in chromosomal complement in *Artibeus* species

**Species**	**C-banding**				
	**Pericentromeric**	**Terminal**	**Interstitial**	**Dispersed**	**Distal**
*Artibeus obscurus*	+	5p, 6p, 7p, 9p	1*, 2q, 5q, 6q	Y_1 _e Y_2_	-
*Artibeus fimbriatus*	+	5p, 6p, 7p	-	Y_1 _e Y_2_	-
*Artibeus lituratus*	+	5p, 6p, 7p	6q	Y_1 _e Y_2_	-
*Artibeus planirostris*	+	5p, 6p, 7p, 9p	1q, 2q, 5q, 6q	Y_1 _	Y_2_

(p) = short arm; (q) = long arm; * = both p and q; + **=** all chromosomes; - = absent

Silver staining (Ag-NORs) showed three pairs of NORs in the terminal region of the short arms in chromosomes 5, 6 and 7 in all species. As a result of remarkable variation in expression and activity, Ag-NORs were counted up to 100 nuclei, which were randomly selected, and the mean number of Ag-NORs per nucleus was determined for each case (Table 2).

**Table 2.** Frequency analyzes of active NORs in the large species of genus *Artibeus*.

**Species**	**Active NOR number per cell**						**Total of cells analyzed**
	**1**	**2**	**3**	**4**	**5**	**6**	
*Artibeus obscurus*	0	17	53	71	12	16	169
*Artibeus fimbriatus*	0	14	40	58	27	22	161
*Artibeus lituratus*	0	7	30	36	22	24	119
*Artibeus planirostris*	0	14	28	47	11	25	125
Total	0	52	151	212	72	87	574
(%) total	0	9.06	26.31	36.94	12.54	15.16	

**Figure 2.**
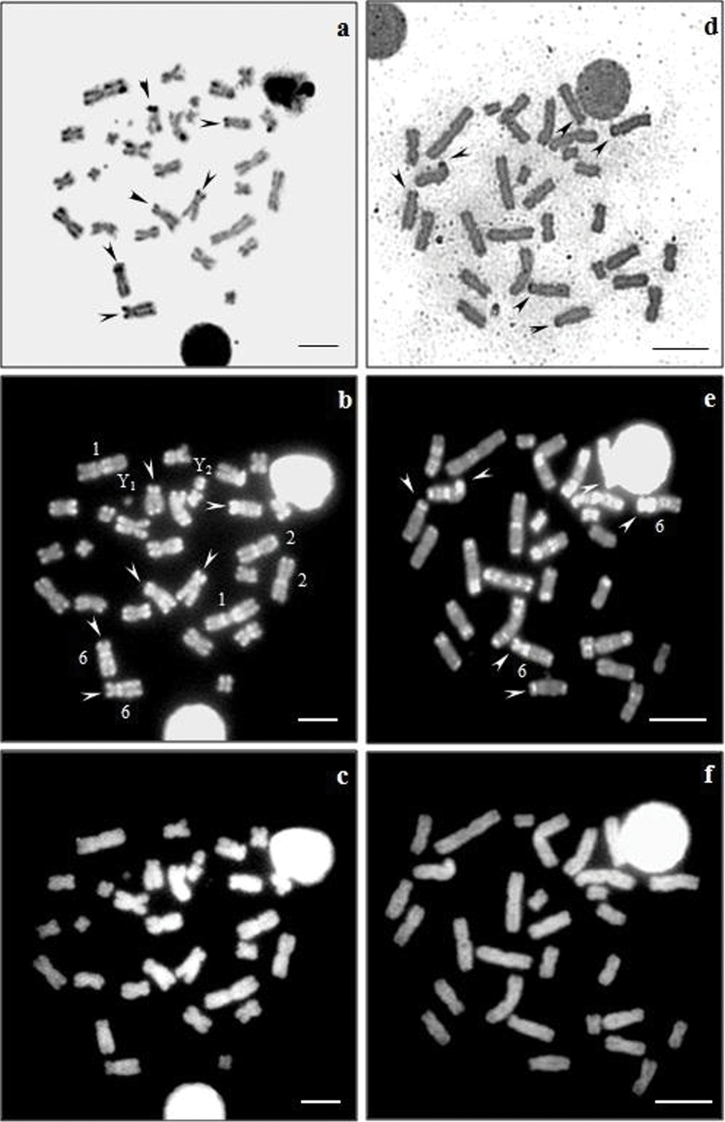
Sequential staining of *Artibeusobscurus* **(a–c)** and *Artibeus fimbriatus* **(d–f)** karyotypes with AgNO_3_/CMA_3_/DA/DAPI. **(a,d)** Ag-NORs, **(b,e)** CMA_3_, **(c–f)** DA/DAPI. Bar = 5 µm.

The sequential staining AgNO_3_/CMA_3_/DA/DAPI showed a correlation between CMA_3 _positive regions and Ag-NORs in the karyotypes of *Artibeus obscurus* and *Artibeus fimbriatus* (Fig. 2a–f). Karyotypes of both species had presented CH blocks associated with Ag-NORs sites, reflecting GC-richness in these heterochromatics clusters. In addition, positive CMA_3_ signals were observed in the pericentromerics regions of certain autosomes, particularly in pairs 1, 2 and 6 of *Artibeus obscurus* and in pair 6 of *Artibeus fimbriatus* (Fig. 2b, e). On the other hand, a uniform pattern was observed in all the chromosomes after DA/DAPI staining (Fig. 2c–d).

## Discussion

Our data regarding diploid number, chromosome morphology and sex determination system obtained for *Artibeus obscurus*, *Artibeus fimbriatus*, *Artibeus lituratus* and *Artibeus planirostris* karyotypes are in agreement with those previously described in the literature (Baker and Hsu 1970, Gardner 1977, Baker et al. 2003). The autosomal complements presented are morphologically similar to other, except by length of the sex chromosomes Y_1_ and Y_2_, which varies from punctiform elements to well-defined acrocentric chromosomes, as firstly described by Hsu et al. (1968) for *Artibeus lituratus*, *Artibeus jamaicensis* Leach, 1821 and *Artibeus toltecus* Saussure, 1860.

The multiple sex chromosome system XY_1_Y_2 _has been widely reported within the genus *Artibeus*, e.g. *Artibeus aztecus* Andersen, 1906, *Artibeus glaucus* Thomas 1893, *Artibeus toltecus*, *Artibeus concolor*, *Artibeus cinereus* Gervais, 1856, *Artibeus hirsutus* Andersen, 1906, *Artibeus inopinatus* Davis et Carter, 1964 and *Artibeus jamaicensis*, and for other 23 species of family Phyllostomidae, as predominant type of sex-determining mechanism in this group (Baker and Hsu 1970, Varella-Garcia et al. 1989, Wetterer et al. 2000, Baker et al. 2003, Noronha et al. 2009). In mammals, this sexual system has been reported in marsupials, insectivores (shrews), carnivores (mongoose), rodents (hamsters) and in artiodactyls (gazelles) (reviewed in Gruetzner et al. 2006). Its origin involves a single sex chromosome-autosome translocation, which in meiosis leads to one sexual trivalent structure formed by XY_1_Y_2_ (Rodrigues et al. 2003, Noronha et al. 2004).

The CH distribution was evaluated and intercompared in the large *Artibeus* and with others phyllostomatids, pointing out an extensive similarity of CH pattern localized in the pericentromeric region (Rodrigues et al. 2000, Barros et al. 2009, Sbragia et al. 2010). Additionally, CH blocks were also found in the terminal region of chromosome pairs 5, 6 and 7 that has been considered a characteristic shared by subfamily Stenodermatinae (Souza and Araújo 1990, Santos and Souza 1998b, Silva et al. 2005).

On the other hand, a particular set of CH blocks was observed in *Artibeus obscurus*, *Artibeus fimbriatus*, *Artibeus lituratus* and *Artibeus planirostris*. This finding allowed the individualization and differentiation of each species for karyotype comparison (Fig. 1). *Artibeus fimbriatus* and *Artibeus lituratus* karyotypes showed a closer CH distribution differing only by one heterochromatin block. Furthermore *Artibeus obscurus* and *Artibeus planirostris* karyotypes presented more interstitial heterochromatin.

The occurrence of intrageneric variation on CH distribution had been described only in sporadic cases among phyllostomatids whose extensive karyotypic conservation is widely known. In turn, the genus *Artibeus* is widely cited as a chiropteran group that exhibits low rate of karyotype evolution whereas: (1) most of species had same diploid number (30/31) and (2) G-banding patterns are essentially identical (Baker and Bickham 1980, Baker et al. 2003).

The other parameter evaluated intercomparison was the NORs localization by silver staining. The Ag-NORs were situated on the subtelocentric autosomes 5, 6 and 7 of all species. The data obtained for *Artibeus lituratus*, *Artibeus planirostris* and *Artibeus fimbriatus*, together with the new data of *Artibeus obscurus*, were similar those described by Santos et al. (2002). These authors employed FISH with 18S ribosomal probe allied to silver staining to investigate the precise localization of rDNA sites, and discovered a non-correlation between the number and distribution of the NORs in *Artibeus cinereus* Gervais, 1856, being the first report on silent NORs in bats. They also had distinguished two rDNA sites patterns for *Artibeus* genus: 1) in the distal regions of the short arms of pairs 5, 6 and 7 (*Artibeus lituratus*, *Artibeus jamaicensis* Leach, 1821 and *Artibeus fimbriatus*) and 2) in the interstitial region of the long arms of pairs 9, 10 and 13 (*Artibeus cinereus*). In addition, *Artibeus fimbriatus* had one NOR in the interstitial region on the long arm of pair 5, that it was not observed in this work, which may indicate a chromosomal polymorphism for this species.

As only active NORs could be visualized in our data, the variation in Ag-NORs activity for cell was also investigated (Table 2). In the most of cells analyzed (> 500), the frequency of active NORs was 3 or 4 black spots (26.31 to 36.94 %). Such variability is in accordance with other studies on a NOR sites activity in Phyllostomidae bats that presents multiple NORs (Morielle and Varella-Garcia 1988, Souza and Araújo 1990, Santos et al. 2002).

The association between NORs and CH by GC-specific fluorochromes staining presented in this work for *Artibeus obscurus* and *Artibeus fimbriatus*, has also been reported to *Artibeus lituratus*, *Artibeus jamaicencis*, *Desmodus rotundus* Geoffroy, 1810, *Diphylla ecaudata* Spix, 1823 and *Lonchorhina aurita* Tomes, 1863. On the other hand, *Carollia perspicillata* Linnaeus, 1758, *Molossus molossus* Pallas, 1766, *Molossus ater* Peters, 1865, *Molossops planirostris* Peters, 1865, *Phyllostomus discolor* Wagner, 1843 and *Trachops cirrhosus* Spix, 1823 NORs and CH were CMA_3_ neutral. The reason for that is probably in heterogeneity of base composition of the intergenic regions related to NORs. In some cases, the triple staining with CMA_3_/DA/DAPI has also enhanced the patterns of R-bands with CMA_3,_ an uniform staining with DA/DAPI or a weak G-banding pattern, as it has been observed in some bat’s families (Santos and Souza 1998a, 1998b, Santos et al. 2001, Leite-Silva et al. 2003, Barros et al. 2009).

## Conclusion

Classical and molecular cytogenetic markers, associated to taxonomic studies, have provided a better understanding of phylogenetic relationships and the mechanisms responsible for chromosomal divergence in the different taxa in the order Chiroptera. Cytogenetic analysis of all Brazilian species of the subgenus *Artibeus* allowed us to reveal the conservative and specific chromosomal features among their karyotypes. Furthermore, it was possible to identify intrageneric and interespecific divergences in a group that up to today has been characterized by showing extensive karyotypic conservation. The cytogenetic techniques herein employed, demonstrated the usefulness of C-banding in the identification and correct individualization of the large *Artibeus* that live sympatrically in the northeastern of Brazil, thus providing an important tool in the cytotaxonomic differentiation of this genus.

## Appendix

**Familia Phyllostomidae**

***Artibeus obscurus***

M 318 (3214) – Saltinho (Rio formoso)

M 319 (3206) – Saltinho (Rio formoso)

M 336 (3235) – Saltinho (Rio formoso)

M 337 (3238) – Saltinho (Rio formoso)

M 340 (3216) – Saltinho (Rio formoso)

M 344 (3237) – Saltinho (Rio formoso)

M 359 (3230) – Saltinho (Rio formoso)

M 381 (3181) – Saltinho (Rio formoso)

M 397 (3186) – Dois irmãos (Recife)

M 455 (3189) – Saltinho (Rio formoso)

M 478 (3179) – Saltinho (Rio formoso)

***Artibeus fimbriatus***

M 346 (3215) – Saltinho (Rio formoso)

M 382 (3184) – Saltinho (Rio formoso)

M 395 (3177) – Dois irmãos (Recife)

M 453 (3175) – Saltinho (Rio formoso)

M 479 (3192) – Saltinho (Rio formoso)

***Artibeus lituratus***

M 221 (3418) – Igarassu

M 379 (3178) – Saltinho (Rio formoso)

M 446 (3191) – Saltinho (Rio formoso)

M 454 (3182) – Saltinho (Rio formoso)

M 475 (3188) – Saltinho (Rio formoso)

M 476 (3180) – Saltinho (Rio formoso)

***Artibeus planirotris***

M 118 (3424) – Igarassu

M 124 (3212) – Igarassu

M 137 (3423) – Aldeia (Camaragibe)

M 188 (3428) – Igarassu

M 262 (3220) – Água Preta (Fazenda Camarão)

M 263 (3218) – Água Preta (Fazenda Camarão)

M 393 (3202) – Dois irmãos (Recife)

M 400 (3176) – Dois irmãos (Recife)

M 401 (3196) – Dois irmãos (Recife)
